# Supplementary material for: Co-infections of Malaria and Geohelminthiasis in Two Rural Communities of Nkassomo and Vian in the Mfou Health District, Cameroon
Source: PLoS Negl Trop Dis. 2014 Oct 16;8(10):e3236. doi: 10.1371/journal.pntd.0003236 (PMC4199518; doi:10.1371/journal.pntd.0003236)
Supplement: Checklist S1 — STROBE checklist. (DOC) [file pntd.0003236.s001.doc]

STROBE Statement—Checklist of items that should be included in reports of ***cross-sectional studies***

|  | Item No | Recommendation |
| --- | --- | --- |
| **Title and abstract** | 1 | (*a*) Co-infections of Malaria and Geohelminthiasis in two rural communities of Nkassomo and Vian in the Mfou Health District, Cameroon. |
|  |  | (*b*) **Background:** Human co-infection with malaria and helmimths is ubiquitous throughout Africa. Nevertheless, its public health significance on malaria severity remains poorly understood. **Methodology/Principal Findings:** To contribute to a better understanding of epidemiology and control of this co-infection in Cameroon, a cross-sectional study was carried out to assess the prevalence of concomitant intestinal geohelminthiasis and malaria, and to evaluate its association with malaria and anaemia in Nkassomo and Vian. From a total of 263 participants aged 1-95 years, finger prick blood specimens were collected for malaria microscopy, assessment of haemoglobin levels, and molecular identification of parasite species by PCR. Fresh stool specimens were collected for the identification and quantification of geohelminths by the Kato-Katz method. The prevalence of malaria, geohelminths, and co-infections were 77.2%, 28.6%, and 22.1%, respectively. *Plasmodium* *falciparum* was the only malaria parasite species identified with mean parasite density of 111 (40; 18,800) parasites/µl of blood. The geohelminths found were *Ascaris* *lumbricoides* (21.6%) and *Trichuris* *trichiura* (10.8%), with mean parasite densities of 243 (24; 3,552) and 36 (24; 96) eggs/gram of faeces, respectively. Co-infections of *A. lumbricoides* and *P. falciparum* were the most frequent and correlated positively. While no significant difference was observed on the prevalence of single and co-infections between the two localities, there was a significant difference in the parasite density of *A. lumbricoides* infection between the two localities. The overall prevalence of anaemia was 42% with individuals co-infected with *T. trichiura* and *P. falciparum* (60%) being the most at risk. While the prevalence of malaria and anaemia were inversely related to age, children aged 5-14 years were more susceptible to geohelminthiasis and their co-infections with malaria.  **Conclusion/Significance:** Co-existence of geohelminths and malaria parasites in Nkassomo and Vian enhances the occurrence of co-infections, and consequently, increases the risk for anaemia. |
| Introduction | | |
| Background/rationale | 2 | In many afro-tropical countries, parasitic co-existence is common with increased potential for co-infection, which may adversely impact the outcome of the diseases they cause. [1]. Of all human diseases caused by protozoan parasites malaria has the greatest burden and is responsible for most deaths amongst young children in sub-Saharan Africa, accounting for 90% of all cases in the world [2]. Until the past decade, intestinal worms have been neglected due to insufficient knowledge of their impact on human life [3-6]. The fact that intestinal worms affect more than two-thirds (70%) of humans has led to growing interest to understand their epidemiology and interactions with other parasitic infections [7, 8]. In Cameroon, both malaria and helminth infections co-exist and are ranked amongst the major cause of parasitic mortality and morbidity. *Plasmodium* *falciparum* is the most prevalent and virulent of the malaria parasites [9-12], while the geohelminths (*Ascaris lumbricoides, Trichuris trichiura*, and Hookworm *ssp*.) and *Schistosoma* *mansoni* are the major helminthic parasites [3, 13-16]. Although there is much literature on the epidemiology of malaria and intestinal worms separately, little is known about the distribution and impact of their co-infections on the population across the country [11, 17]. Due to the differences in the physiological, anthropological, genetic, immunological or geo-ecological factors, infections with multiple parasite species may not necessarily be independent within an individual, and could result in positive or negative associations in disease manifestation. The implications of concomitant malaria and helminth infections have been mainly explored and indicate that their interactions can decrease the course of malaria infection and disease [18, 19]. On the other hand, co-infected school-aged children have been shown with different phenotypes in the pathogenesis of malaria with increasing risk of developing severe malaria [1, 10, 18, 20-22]. These conditions could further result in increase malaria parasitaemia, increased risks of anaemia and malnutrition, modification of the immune response to malaria [12, 23, 24], and increased mortality. Therefore, the acquisition of requisite knowledge on co-morbidity and interactions between malaria and helminthiasis would be highly invaluable to controlling malaria infection and clinical disease. The control of these parasitic diseases in Cameroon rely on the WHO strategies, which lay emphasis on a combined control approach for malaria infection [11] and deworming activities for helminth parasites [17]. However, implementation of such measures requires essential requisite information on the geo-ecological distribution and efficiency in infection transmission. Thus, this study sought to determine the prevalence of malaria and geohelminths co-infection and evaluate its impact on malaria and anaemia in Nkassomo and Vian, two rural communities of Cameroon. The findings from the study will provide useful information necessary to design strategies for effectively controlling and managing malaria in the context of co-infection. |
| Objectives | 3 | - Determine the prevalence of geohelminthiasis, malaria, and their co-infection in the study population. - Establish correlation/association between geohelminth and malaria parasite densities in co-infected patients. - Determine the prevalence of anemia and its correlation with age, fever and parasite densities in the study population. |
| Methods | | |
| Study design | 4 | This study received ethical and administrative authorizations respectively from the IMPM institutional ethics review committee and the competent authorities of the Mfou health district. Participation was strictly voluntary and was dependent on informed written consent by the participant or assent by the parent/guardian for children. All positive cases for malaria and/or intestinal worms were treated for free according to the Cameroonian anti-malarial and anti-helminth treatment guidelines and if necessary referred to the Mfou district hospital for appropriate management. A single dose of Paracetamol and iron tablets was respectively given to participants with fever or anaemia. |
| Setting | 5 | The study was carried out in two rural communities (Nkassomo and Vian) of the Mfou health district (4^o^27` N and 11^o^38` E), a forest area located in the Mefou-Afamba division, in the Centre region of Cameroon. The climate is typically equatorial with two discontinuous dry and wet seasons. The annual average rainfall measures 2,000 mm with an annual average temperature of 24°C. The population is made up of 71,373 inhabitants (4,100 in Nkassomo and 3,248 in Vian) with about 82.7 inhabitants/km². Mfou is a multiethnic community made up of the Ewondo, Bané and Tsinga, with farming being the main economic pursuit. Commonly grown crops include cassava, maize and oil palm [25]. Houses are built in semi-dur with crevices and open joints serving as hide outs for mosquitoes. These villages lack access to potable water. Toilet facilities made up essentially of pit latrines, which are generally poorly constructed and insufficient for the members of each household. It is common to find pools of standing and dirty water close to some households that are prolific for mosquito. |
| Participants | 6 | (*a*) The study was conducted from February to March 2011 during the long dry season in the Mfou health district. Samples were collected from each household and only from individuals who willingly accepted to participate and provided written and signed informed consent and have lived in the village for at least the past six months. A total of 263 participants from the two rural communities (131 from Nkassomo and 132 from Vian) aged 1 to 95 years old took part in the survey and were divided into 6 groups as follows: pre-school children (<5 yrs), young school children (5-9 yrs), old school children (10-14 yrs), adolescents and young adults (15-24 yrs), adults (25-49 yrs), and aged people (≥ 50 yrs). Each participant was clinically examined by a medical doctor for febrile symptoms (fever, axial T ≥ 37,5^o^C) or any other clinical conditions (headache, abdominal discomforts, etc.), and information on age and sex taken prior to specimen collection. |
| Variables  Data sources/ measurement | 7-8* | The age, sex, and axial temperature of each participant were documented to help in the classification of the different infections.  **Blood collection and Processing**  Finger prick blood samples collected in heparinised micro-capillary tubes were used to prepare thick and thin smears for malaria microscopy, blot filter papers (Whatmann #3) for *Plasmodium* DNA isolation and PCR amplification for species determination, and measure the haemoglobin level.  **Detection and quantification of malaria parasites.** The thin and thick blood smears air-dried were fixed in 75% methanol and 50% May-Grunwald (thin film only) and stained using 10% Giemsa solution for both thin and thick films. Slides were then microscopically examined for the presence of malaria parasites by two independent microscopists. Where parasites were found the parasite density was determined by counting the number of parasites against 200 leucocytes. The counts were expressed as the number of parasites per micro litre of blood (p/µl), assuming an average leukocyte count of 8,000 cells/µl of blood [26]. For every slide where the difference in parasitaemia between the two readers was greater than 5 p/µl of blood, a third reader had to re-examine the slide and the mean of the two closest values considered.  ***Plasmodium DNA extraction and amplification.*** Fresh blood specimens from heparinised micro-hematocit tubes were transferred onto Whatmann #3 filter paper and used for the extraction of Plasmodium DNA and subsequent PCR amplification for the identification of Plasmodium species. [27-29].  **Assessment of anaemia.** A drop of capillary blood was set down on a strip of a portable haemoglobinometer (URIT MEDICAL ELECTRONIC Co, LTD, 2008: URIT-12; < 58g; 102mmx50mmx19mm; DC 3V; 8 years) to determine the total haemoglobin level according to the product instructions. Anaemia was considered as total haemoglobin level < 11g/dl of blood and classified according to the WHO definition as either mild, moderate or severe [30].  **Stool collection and processing**  Prior to stool sampling, clear instructions and clean well labelled stool collection vials were given to each participant for proper stool collection. Stool smears were prepared using the Kato Katz technique for the morphological identification of geohelminth eggs for *A. lumbricoides, T. trichiura, and* Hookworms *spp*., or larval stage for *S. stercoralis* [31]. The smears were made on duplicate slides per participant, kept refrigerated overnight and then microscopically examined within 7-9 hrs to avoid missing hookworm eggs and minimize bias on the parasitological data. Helminth eggs were counted in 41.7 mg of stool and counts were extrapolated as the number of eggs per gram of stool (eps). |
| Bias | 9 | Two blood and two stool slides were prepared per participant and examined by two independent microscopists to minimize the measurement bias on the parasitological data. In addition, stool slides were refrigerated overnight and examined microscopically within 7-9 hrs after Kato Katz smear preparation to avoid missing hookworm eggs and minimizing the measurement bias on the parasitological data. The nested PCR method was carried out in addition to microscopy to confirm the *Plasmodium* malaria species found. As this a cross-sectional study, further epidemiological studies are needed to support the finding obtained. |
| Study size | 10 | The study addressed all the population of Nkassomo and Vian localities. Based on the inclusion and non-inclusion criteria and also on the availability of the population of these localities, 263 volunteers fully agreed to participate in the study and signed the informed consent form. |
| Quantitative variables | 11 | Quantitative values were expressed as geometric mean value (minimum value; maximum value) or arithmetic mean value ± Standard Deviation (SD). Qualitative values were expressed in frequency or percentage. |
| Statistical methods | 12 | (*a*) Statistical analysis of the data was performed using SPSS 18.0 software (SPSS Inc, Chicago IL, USA). Threshold for statistical significance was set at *P <0.05*. Chi-square test and One Way ANOVA were used to set the difference in proportions and means respectively. |
|  |  | (*b*) Pearson logistic regression test was used to establish the correlation between variables (*Plasmodium* densities, geohelminth densities, fever, and anaemia).  Prevalence was defined as the proportion of individuals found harbouring the parasite in question to the sum total of the study population. Co-infection was defined as the simultaneous presence of at least one helminth parasite species plus malaria parasite in the same host, and was classified as either single or mixed, depending on the number of parasites found. |
| Results | | |
| Participants | 13* | (a) Overall 263volunteers participated in the study after signing an informed consent form. |
| Descriptive data | 14* | (a) Of the 263 study participants 131 (49.8%) were from Nkassomo and 132 (50.2%) from Vian. Of these, there were 123 (49.6%) males and 139 (53.1%) females. The median age in the study population was 26.1 ± 22.4 yrs (range, 1 to 95 yrs). Based on the WHO criteria, the median axial temperature was 37.3 ± 0.5°C (range, 35.5 to 41 °C) and 91 (34.6%) participants had fever. Although children in the age group of 5-9 yrs had the most cases of fever no difference was observed based on age, sex and locality. A total of two hundred and eighteen participants (82.9%) was found to carry at least one of the identified parasites (*Plasmodium falciparum, A. lumbricoides* or *T. trichiura*). |
|  |  | (b) Indicate number of participants with missing data for each variable of interest:  Sex (01), Age (07). |
| Outcome data | 15* | Three outcome events:  Malaria and geohelminthiasis epidemiology,  Co-infection epidemiology (among co-infected individuals),  Distribution of anaemia (among non-infected, single and co-infected individuals). |
| Main results | 16 | (*a*) **Parasite prevalence and intensities**  The overall prevalence of malaria was 77.2% (n = 203) with *Plasmodium falciparum* being the only parasite species found. The mean parasite density in the population was 111 (40; 18,800) parasites per micro litre of blood (P/µl). Based on the study locality, the prevalence of malaria in Nkassomo was 78.6% (n = 103) and 75.8% (n = 100) in Vian. This did not vary significantly in the prevalence. A similar trend was observed for the parasite density with an average parasite load of 125 (40; 14,760) P/µl in Nkassomo, and 98 (40; 18,800) P/µl in Vian. Sex did not influence the prevalence and intensity of *P. falciparum* in both localities. Although all age groups were affected, the peak of malaria prevalence was observed in children less than 9 years old. Only 32.5% (n = 66) of malaria positive cases had fever and with the highest parasite densities (Tables 1 and 2).  *Ascaris lumbricoides, Trichuris trichiura* were the only geohelminth parasites detected. Of the 66 (28.6%) participants positive for geohelminth infection, there were 50 (21.6%) *A. lumbricoides*, 25 (10.8%) *T. trichiura.* However, there were 9 (3.9%) cases with both infections. The geometric mean of the parasite density for each species was 243 (24; 3,552) eggs per gram of faeces (eps) for *A. lumbricoides,* and 36 (24, 96) eps for *T. trichiura*. There was no observed significant difference in the prevalence of the two parasites between the two localities. However, the parasite densities were higher in Vian with a significant difference observed for *A. lumbricoides* (p = 0,001). With regards to sex and age, the prevalence of *A. lumbricoides* was similarly distributed and was higher in children between the ages of 5 years and 14 years (p = 0.002), whilst no difference was observed for *T. trichiura* infection between the males and the females (p = 0,071) and by age groups. Overall, school-aged children (5-14 yrs) had the highest prevalence and parasite density of geohelminth infections. However, only the prevalence was significantly different when compared to other age groups (p = 0.008) (Table 1).  With regards to malaria and geohelminth co-infection, a total of 51 (22.1%) participants were infected with at least one geohelminth parasite and *P.* *falciparum*. Two cases of co-infections were observed and classified as either single co-infections of *A. lumbricoides* and *P. falciparum* (38 [16.5%]), and of *T. trichiura* and *P. falciparum* (20 [8.7%]); or mixed co-infections of the three parasites (7 [3.0%]). As observed with single infections of individual parasites, there was significant difference in the prevalence of co-infections between the two study localities. Co-infections with *T. trichiura* and *P. falciparum* was observably higher in males than females (p = 0.009). Similarly the overall co-infection was also higher in males than females (p = 0.025). The highest prevalence of co-infection was observed in children between 5 and 14 years old (p = 0.010). With regards to the parasite densities, single infections with only *A. lumbricoides* (179 [24; 1,920] eps) and only *P. falciparum* (107 [40; 4,000] p/µl) had generally lower parasite densities compared to when the two occurred as co-infections of *A. lumbricoides* (257 [24; 3,552] eps, p = 0.012) and *P. falciparum* (143 [40; 2,360] p/µl, p = 0.018). Although there was a similar observation for mixed co-infections for *A. lumbricoides*, *T. trichiura* was associated with low densities of *P. falciparum*, but the difference was not significant (Table 3). On the other hand, logistic regression analysis (Figure 2) showed a positive association between the parasite densities of *A. lumbricoides* (independent variable) and *P. falciparum* (dependant variable) in co-infected individuals (R = 0.406, Correlation coefficient = 0.381, p = 0.175).  **Anaemia and infections**  According to the WHO classification, out of the 263 participants in the study, 110 had haemoglobin levels less than 11 g/dl of blood, giving an overall anaemia prevalence of 42%. However, most the anaemic cases were classified as moderate anaemia with a mean haemoglobin level of 9.8 ± 0.8 g/dl. Only one case of severe anaemia (0.9%) was observed with a total haemoglobin level of 6.7 g/dl. Fifty three (40.5%) and 57 (43.5%) participants were found to be anaemic in Nkassomo and Vian, respectively. Although anaemia did not differ by locality and sex, the prevalence of anaemia decreased significantly with the age groups (p = 0.003) (Table 1).  Overall, all anaemic cases were found to carry at least one of the detected parasitic infections. There were 13 (52%), 95 (46.8%), and 19 (38%) cases of anaemia amongst those infected with *T. trichiura*, *P. falciparum*, and *A. Lumbricoides,* respectively. The prevalence however was observed to increase by up to 71% in co-infection cases (Figure 1). Although anaemia was more frequent in those infected with *T. trichiura* and *P. falciparum* as co-infections, *A. lumbricoides* did not show any influence on the prevalence of anaemia in co-infections. Exploratory multiple linear regression analysis depicted positive associations between the parasite densities (*P. falciparum* and *T. trichiura*) considered as the independent variables with anaemia and fever, considered as the dependent variables. However, these infections were not significant predictors of anaemia and fever in the area. |
| Other analyses | 17 | Correlation between parasite densities and age groups,  Correlation between haemoglobin levels and age groups in infected participants,  Correlation between parasite densities and haemoglobin levels. |
| Discussion | | |
| Key results  Limitation  Interpretation  Generalisability. | 18 | Results of this study provided preliminary data on the epidemiology of malaria and geohelminthiasis in Nkassomo and Vian, two rural communities of the Mfou health district in the Centre region of Cameroon. Malaria in these communities is high and mainly due to *Plasmodium falciparum*. The overall observed prevalence of 77.2% is higher than the 40.6% recorded in 2009 amongst schoolchildren in the same health district [12]. This difference is not unlikely since our study population was larger. This observed prevalence is equally higher than those obtained in other regions of the country and elsewhere [7, 9, 22, 32] and could be linked to differences in the geoecological and climatic conditions that might influence malaria vector breeding and distribution in different areas. Although the mean intensity of *P. falciparum* malaria can reach the severe level (>5,000 p/µl) in most regions where malaria is endemic [10], our study area revealed a very low parasite densities (< 500 p/µl). Malaria intensity was therefore qualified as light according to Trape *et al*. [33] classification, and this could be attributed to the difference between the period of infection and the survey, the infection rate, and the preventive measures (chemoprophylaxis or auto-medication at individual level) considered as the main factors that can influence the infection intensity [34]. Although malaria is usually shown with highest prevalence among children and pregnant women, our study revealed that pre and young schoolchildren (0-9 yrs) had the highest prevalence and intensities of malaria, which did not differ significantly amongst age groups. This is probably due to hyper transmission of the infection in this locality and the acquisition of plasmodial immunity with increasing age after 9 years [2, 11, 32]. In fact, as sated previously, parasitic distribution is generally not homogenous and malaria can affect the age with which adequate immunity is acquired when transmission level is important [7]. Similar results have been obtained in a previous study in Ghana by Ehrhardt *et al*. [35]. The environmental and sanitary conditions in Nkassomo and Vian are prolific to the development of *Anopheles* mosquitoes and consequently to the development and propagation of *Plasmodium* parasite for which clinical diagnosis and non-compliance to confirmed diagnosis remain a subject of case study. In fact, this study revealed that only 33% of participants positive to *Plasmodium falciparum* were symptomatic to malaria. This low symptomatology in malaria could be attributed to low parasitic intensities of *P. falciparum* and probably the carriage of *Ascaris lumbricoides,* as most participants infected with both *P. falciparum* and *A. lumbricoides* were significantly asymptomatic to malaria (Table 2). Also, *A. lumbricoides* can curtail the symptoms and the course of malaria infection and disease [19, 21, 22]. Even though the parasite density of malaria in the region was very low, the observed hyperendemicity is indicative that efforts should be intensified in controlling malaria in Mfou. Preceding this study, the government of Cameroon implemented a large scale free distribution of long lasing insecticidal nets to all households in order to curb the malaria burden. Further studies would be carried out to evaluate the impact of this intervention on the disease burden and transmission.  Helminth parasites are highly prevalent in rural communities due to poor sanitary conditions prevailing in most of these areas [8]. In order to assess the prevalence of these infections in Mfou rural areas, our study recorded an overall prevalence of 28.6% for geohelminth infections, with *A. lumbricoides* as the main parasite species, followed by *T. trichiura*. Although similar findings were obtained for earlier studies on schoolchildren in the same region in 2009 [12], it did not corroborate with those in the South-West region of the country and Ethiopia [1, 15]. This observation is not uncommon as helminths` distribution is generally known to vary with environmental conditions that are directly related to the level of healthiness of each area [13, 36]. No case of any other geohelminths (Hookworms *spp*., *S. stercoralis*) was found. However, as hookworm ova is likely deform and may clear within 24 hours following Kato Katz smear preparation [31], the absence of Hookworms *spp.* could stand as a possible bias for this study due to the time spent (7-9 hours) between the preparation and examining of the Kato Katz smears. Knowing that *A. lumbricoides* infection is usually associated with higher parasite intensity in contrast to *T. trichiura* infection, and according to the WHO classification of helminth parasite densities [37], this study revealed that both parasites are characterized by light intensity in Mfou. This low intensity and prevalence of intestinal parasites in Mfou rural areas could be attributed to the multiple deworming campaigns that the government implemented in the region the year before this study [17]. In addition can be the chemoprophylaxis applied at individual level as preventive measure. This auto medication seemed to be important in Vian where the intensities of *A. lumbricoides* were significantly higher compared to Nkassomo. The proximity of Nkassomo to the lone health facility in the health area could as well be central to this observed difference as there is decreased access of the population of Vian to appropriate drugs in case of infection. Similar results have been observed in two connected villages in previous studies by Nkengazong et al. [15]. However, the infection rate of these species did not differ by locality. Although males were more vulnerable to *T. trichiura* infection, *A. lumbricoides* infection was significantly different amongst age groups with school children (5-14 yrs) having the highest prevalence of *Ascariasis* (p = 0.002) and total geohelminth infection (p = 0.008). This shows that geophagia or soil ingestion is more important in males and school children who are usually in contact with the soil, the main source of contamination for soil-transmitted helminths or geohelminths. Similar results were observed by Hamit et al. [38] and Tchinda et al. [12]. On the other hand, the World Health Organization reported that 70% of total geohelminth morbidity could be avoided when treating only school children (WHO, 2004). To achieve this, sanitary education of the population is crucial. Any control or deworming strategy of geohelminths should therefore be preceded by a full sensitization and awareness of the population about individual hygiene, which is key to any preventive measures against these infections.  So far, very little is known about the distribution and impact of intestinal worm co-infections across Cameroon. This study revealed that many malarial patients were infected by at least a geohelminth parasite with overall prevalence of 22.1%. Observably, *P. falciparum* and *A. lumbricoides* were the main parasite species for malaria and geohelminthiasis and constituted the main co-infection. This was followed by *T. trichiura-P. falciparum* co-infection and mixed co-infection. Our data showed that co-infection had the same distribution with single infections between localities, and that increasing endemicity of single infections is likely to enhance of the establishment of co-infections. In fact, schoolchildren aged 5-14 years had significantly higher prevalence of co-infection (p = 0.001), due of increased susceptibility of this group to *A. lumbricoides* as reported elsewhere [1, 12, 16, 22, 39-41]. In contrast to single infections, association between *T. trichiura* and *P. falciparum* was significantly higher in males due to increased susceptibility of men to *trichinosis* [38]. Thus, treatment of cases of co-infections should rely on the association of measures used to fight against single infections [22].  Co-infected parasites do not necessarily act independently as a consequence of their co-existence in the same host. Indeed, the increase intensities of *P. falciparum* and *A. lumbricoides* in their co-infection was significant. However, akin to earlier studies, the observed influence of *A. lumbricoides* on *P. falciparum* infection in this study was important though not statistically significant due to a decrease in anti-malarial immunity by concomitant helminth infections [1, 19, 20, 21, 42,]. It has also been shown that the risk of developing severe malaria increases in children co-infected with *Ascariasis* due to an increase in malaria intensity. However, the mechanisms of such interactions remain unknown. In contrast to *A. lumbricoides*, *T. trichiura* parasite was associated with a decrease in intensity of *P. falciparum* at a non significant level. Nevertheless, interactions between *T. trichiura and P. falciparum* seemed be higher than those between *A. lumbricoides* and *P. falciparum* in the mixed co-infection when compared to single co-infections (Table 3). A direct consequence of such interactions was observed in the distribution of anaemia amongst the study population.  Overall, all cases of anaemia were carriers of at least one infection with co-infection being the main parasitic factor of anaemia, followed by single infections of *T. trichiura*, *P. falciparum,* and *A. lumbricoides*. The intensity of anaemia was respectively lower and higher than that obtained by Ehrhardt et al. [35] and Degarege et al. [1], who attributed this to the above parasites including Hookworms *spp*. However, it is important to note that these are interesting factors of anaemia in the study area but not probably the only. Further studies should consider other factors as other infections (e.g., Schistosomiasis, Ankylostomiasis), nutritional status, socio-economic level, etc. Anaemia did not significantly differ by locality and sex, but total haemoglobin level increased with age, with children less than five years having the highest prevalence of anaemia probably due to their high infection rate for *P. falciparum* and *T. trichiura* parasites. However, age was not a predictor of anaemia in our study area in contrast to Nkuo-Akenji et al. in South-West Cameroon [10]. According to above observations, this decrease in prevalence of anaemia by age could be linked to acquisition of adequate immunity against responsible infections [23]. The prevalence of anaemia was observed to increase in co-infections compared to single infections, probably due to interactions between *T. trichiura* and *P. falciparum*; because *A. lumbricoides* does not seem to have any impact on haemoglobin level in co-infections as reported by Kouontchou at et al. [4]. This increase in prevalence of anaemia in co-infections is due to additive effects in the mechanisms of these infections on total haemoglobin concentration. In fact, *T. trichiura* is associated with chronic loss of blood and iron in gastrointestinal tube, while *P. falciparum* is associated with bursting and loss of red blood cells during its blood stage, all contributing to haemoglobin loss and anaemia. However, unlike in the southwest region of the country, the intensities of these parasites were not predictors of anaemia in Nkassomo and Vian, [10]. |
| Limitations | 19 | Se above in key results. |
| Interpretation | 20 | See above in key results. |
| Generalisability | 21 | See above in key results. |
| Other information | | |
| Funding | 22 | This project received financial support from the direction of IMPM through the project titled « *Enquête épidémiologique sur le paludisme et les infections gastro-intestinales dans le district de santé de Mfou* » at the Medical Research Centre of IMPM; and the MR4, that provided PCR primers for molecular identification of *Plasmodium* species at the Biotechnology Centre of University of Yaoundé I, Cameroon. |

*Give information separately for exposed and unexposed groups.

**Note:** An Explanation and Elaboration article discusses each checklist item and gives methodological background and published examples of transparent reporting. The STROBE checklist is best used in conjunction with this article (freely available on the Web sites of PLoS Medicine at http://www.plosmedicine.org/, Annals of Internal Medicine at http://www.annals.org/, and Epidemiology at http://www.epidem.com/). Information on the STROBE Initiative is available at www.strobe-statement.org.
